# Temporal trends in hospice deaths and causes of death in Italy, 2011–2022: A nationwide population-based study

**DOI:** 10.1017/S1478951526102193

**Published:** 2026-04-13

**Authors:** Edoardo Varratta, Angela Iurlaro, Giada Minelli, Arianna Guaita, Myriam Macaluso, Maria Beatrice Zazzara, Graziano Onder

**Affiliations:** 1 Università Cattolica del Sacro Cuore Facoltà di Medicina e Chirurgia, Rome, Italy; 2 Istituto Superiore di Sanita, Rome, Italy; 3 Fondazione Policlinico Universitario Agostino Gemelli IRCCS, Rome, Italy

**Keywords:** Hospice, cancer, cardiovascular disease, end of life, palliative care

## Abstract

**Objective:**

Hospices represent the cornerstone of modern palliative services. However, population-level data on hospice utilization and characteristics of patients dying in hospice remain limited to examine national temporal trends in hospice deaths in Italy from 2011 to 2022, with a focus on the underlying causes of death.

**Methods:**

We performed a nationwide, population-based retrospective study using official mortality data from the Italian National Institute of Statistics. All deaths registered in Italy between 2011 and 2022 were included. Hospice deaths were identified as those occurring in licensed hospice facilities.

**Results:**

Hospice beds increased from 1,681 in 2011 to 3,419 in 2022, while hospice deaths more than doubled from 19,179 (3.2% of all deaths) to 43,972 (6.2%). The mean age of hospice deaths rose from 74.0 to 76.6 years. Among patients dying in hospice, neoplasms remained the leading cause of death but declined from 87.0% in 2011 to 73.8% in 2022, while cardiovascular deaths increased from 6.2% to 9.5%, neurological from 1.2% to 3.4%, and respiratory from 1.0% to 2.5%. The proportion of national neoplasm deaths occurring in hospice reached approximately 20% in 2022. Similarly, the proportion of non-neoplasm hospice deaths tripled (0.6–2.1%).

**Significance of the results:**

Between 2011 and 2022, hospice deaths in Italy more than doubled, reflecting substantial progress in expanding access to palliative care. The gradual increase in non-neoplasm hospice deaths suggests a shift toward greater inclusivity, although neoplasm remains predominant.

Key statements

- Hospice care represents the cornerstone of Italy’s palliative-care network, yet national data on temporal trends have been lacking.
- Between 2011 and 2022, hospice deaths more than doubled, with a progressive rise in non-cancer causes such as cardiovascular and neurological diseases.
- These findings indicate major progress in hospice capacity and inclusivity, while highlighting the ongoing need for equitable, diagnosis-independent access across all regions.

## Introduction

In 2010, Italy became one of the first European countries to formally recognize access to palliative care and pain management as universal rights through Law No. 38/2010 (Italian Law 38/2010 2010). This law established the foundation for an integrated, nationwide palliative-care network. Hospices, providing short-term inpatient management for complex symptoms and psychosocial distress, are fully funded by the National Health Service and represent the cornerstone of this network (Italian Law 38/2010 2010; Agenzia Nazionale per i Servizi Sanitari Regionali (AGENAS) 2023). More than 15 years after the adoption of this law, substantial regional disparities persist in bed availability, referral pathways, and integration with community and hospital-based palliative-care teams (Cocchi et al. 2024; Volonnino et al. 2024). In addition, national data on who dies in hospice and how patients’ characteristics have changed over time remain limited, making it difficult to assess equitable access across baseline conditions and diseases.

National mortality data reveal that diseases of the circulatory system remain the leading cause of death in Italy, followed by neoplasms and respiratory and infectious diseases (Istituto Nazionale di Statistica (Istat) 2024). While neoplasm mortality has shown a modest decline over the past decade, chronic non-communicable diseases continue to dominate mortality patterns (Istituto Nazionale di Statistica (Istat) 2024). As palliative care is effective across a wide range of serious illnesses (Kavalieratos et al. 2016; Hughes et al. 2023), the distribution of diagnoses among hospice deaths should reflect that of deaths in the general population.

Understanding how hospice deaths have changed over time helps assess the progress of Italy’s palliative care system toward fair, diagnosis-independent end-of-life care. This study describes national trends in hospice deaths, focusing on underlying causes of death and their alignment with overall mortality patterns.

## Methods

We analyzed national mortality data from the Italian National Institute of Statistics (Istat) for the period 2011–2022. All deaths registered in Italy during this timeframe were included. Causes of death were derived from the national Cause of Death registry, which collects copies of death certificates completed by the medical certifiers for all deaths occurring in Italy. All causes reported on the death certificate are classified according to the International Classification of Diseases, 10th Revision (ICD10) (World Health Organization 2016), using the semi-automated coding system Iris (www.iris-institute.org, accessed on February 25, 2026), which attributes ICD codes for approximately 80% of cases; the remaining 20% are reviewed by expert personnel. For the aim of this study, causes of death were classified into neoplasms (C00–D48), cardiovascular diseases (I20–I25; I60–I69; I10–I15; I30–I52; I70–I99), central nervous system disorders (G00–G99), respiratory diseases (J00–J99), and other conditions.

Death in hospice was defined as death occurring in a licensed hospice facility, as reported in Istat records. In Italy, hospice care is provided by the National Healthcare System (Sistema Sanitario Nazionale), which fully covers the costs of care. In line with a former publication (Zazzara et al. 2023), data on hospice beds in the country were obtained from the Statistical Yearbook of the National Health Service (Italian Ministry of Health 2023).

For each year, we extracted the absolute and relative numbers of hospice deaths, the causes, and the demographic characteristics of hospice deaths, including mean age and sex distribution. Additional analyses were performed to examine differences by sex and cause of death (neoplasm vs. non-neoplasm), comparing hospice deaths with the corresponding total deaths in the national population. Descriptive analyses were conducted to assess temporal trends, expressed as crude numbers, proportions, and mean values. Results were presented graphically to illustrate changes in hospice utilization and cause-specific mortality over time.

## Results

### Hospice bed availability and deaths

Table 1 presents the temporal trends in hospice bed availability and hospice deaths in Italy. The number of hospice beds increased progressively from 1,681 in 2011 to 3,419 in 2022. In parallel, hospice deaths more than doubled, rising from 19,179 (3.2% of all national deaths) in 2011 to 43,972 (6.2%) in 2022, for a cumulative total of 405,681 deaths over the study period. Growth was steady until 2019, when hospice deaths peaked at 43,660 (6.9%). This was followed by a decline in 2020–2021, in association with the COVID-19 pandemic, and then a new increment was seen in 2022. Over time, the mean age at death in hospice rose from 74.0 years (SD 12.3) in 2011 to 76.6 years (SD 12.5) in 2022. The proportion of women also increased modestly, from 46.8% in 2011 to 48.5% in 2022.

**Table 1.** Hospice bed availability and characteristics of hospice deaths in Italy, by year

Year	Hospice beds	Hospice deaths characteristics		
		Number (% of all national deaths)	Age	Women
			(mean ± SD)	(%)
2011	1,681	19,179 (3.2)	74.0 ± 12.3	46.8
2012	2,069	28,642 (4.7)	74.2 ± 12.5	47.7
2013	2,307	31,862 (5.4)	74.2 ± 12.3	46.6
2014	2,551	34,029 (5.7)	74.4 ± 12.3	46.4
2015	2,567	35,801 (5.6)	74.7 ± 12.3	46.8
2016	2,693	37,744 (6.1)	75.0 ± 12.3	46.6
2017	2,777	39,232 (6.1)	75.1 ± 12.3	46.5
2018	3,138	41,095 (6.5)	75.4 ± 12.4	46.8
2019	3,382	43,660 (6.9)	75.8 ± 12.3	47.3
2020	3,236	38,844 (5.2)	76.0 ± 12.5	48
2021	3,151	39,621 (5.6)	76.4 ± 12.4	48.2
2022	3,419	43,972 (6.2)	76.6 ± 12.5	48.5

## Causes of death

**Figure 1.**
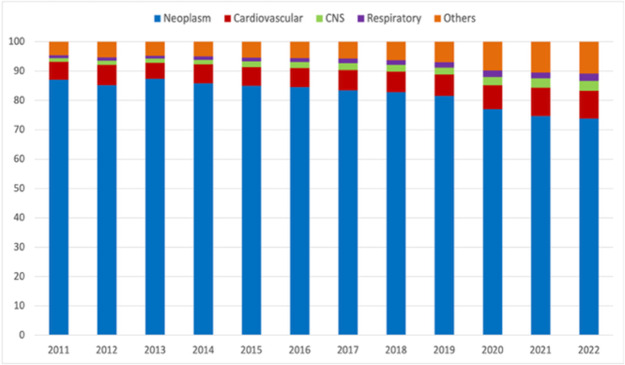
Proportion of hospice deaths by cause (neoplasms, cardiovascular diseases, central nervous system disorders, respiratory diseases, and other conditions), relative to all hospice deaths in Italy, 2011–2022.

As shown in Figure 1, neoplasms consistently represented the leading cause of hospice deaths. However, the proportion of deaths due to neoplasms as compared with the overall number of deaths occurring in hospice decreased from 87.0% in 2011 to 73.8% in 2022. During the same period, the proportion of deaths due to cardiovascular diseases rose from 6.2% to 9.5%, those due to central nervous system disorders from 1.2% to 3.4%, respiratory diseases from 1.0% to 2.5%, and other conditions from 4.7% to 10.9%.

**Figure 2.**
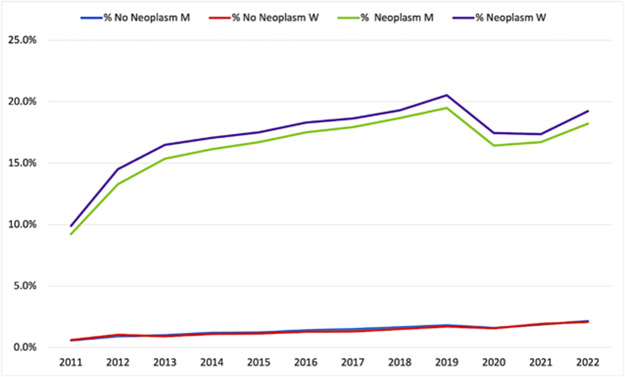
Hospice deaths as a proportion of total deaths in the Italian population, stratified by gender and cause (neoplasm vs. non-neoplasm), 2011–2022.

Figure 2 presents hospice deaths as a proportion of all deaths in the Italian population, stratified by gender and cause (neoplasm vs. non-neoplasm). Between 2011 and 2019, the proportion of deaths due to neoplasms occurring in hospice increased steadily, reaching nearly 20% for both men and women. This trend reversed in 2020–2021, with a sharp decline, and only partially recovered in 2022, remaining below the 2019 peak. By contrast, non-neoplasm hospice deaths, although much fewer in absolute numbers, more than tripled over the study period, rising from approximately 0.6% in 2011 to 2.1% in 2022 in both sexes. Notably, non-neoplasm hospice deaths showed a more stable trajectory during the pandemic years compared with neoplasm deaths.

## Discussion

This study, covering more than 400,000 hospice deaths between 2011 and 2022, provides a comprehensive picture to date of hospice utilization in Italy. The results show a progressive increment in both hospice capacity and use, with deaths in hospice more than doubling over the decade. This growth, accompanied by a progressive aging of the hospice population and a modest increase in women, suggests broader access for older, frailer patients and highlights hospice care’s growing importance within Italy’s health system across all care settings (Beccaro et al. 2007; Volonnino et al. 2024).

The Italian trajectory mirrors that of other high-income countries that have progressively embedded hospice into their end-of-life care systems. In England, for instance, the proportion of deaths occurring in hospice rose from around 3% in the early 1990s to nearly 7% by 2012, reflecting the gradual normalization of hospice as a mainstream component of end-of-life provision (Sleeman et al. 2016). Comparable increases have been reported in Australia (Khalil et al. 2020), the United States (Teno et al. 2018; Cross et al. 2021), and Canada (Qureshi et al. 2018), although the pace and pattern of growth vary according to national models of funding and referral. By 2022, hospice accounted for 5.2% of all deaths in Italy, a figure broadly consistent with other European countries where hospice remains primarily an inpatient service (van Steijn et al. 2021).

Two main changes emerge: a drop in hospice deaths during the COVID-19 pandemic, reflecting its impact through fewer referrals, admission limits, staff redeployment, and more home deaths, and a shift in the diagnostic profile of patients who died in hospice. While neoplasms remain by far the leading cause of hospice death, their relative share fell from nearly 90% in 2011 to less than 75% in 2022, accompanied by parallel increases in cardiovascular, neurological, and respiratory diseases. This shift represents an encouraging, though still incomplete, diversification of the hospice population. The Italian data therefore suggest that hospice eligibility is gradually broadening beyond oncology, reflecting growing awareness of palliative-care needs in non-neoplasm conditions. Nevertheless, patients with advanced heart failure, chronic lung disease, dementia or neurodegenerative disorders remain substantially under-represented, despite experiencing comparable levels of symptom burden, functional decline, and caregiver distress (Tobin et al. 2022).

A key strength of this study is that it offers a national overview of hospice use in Italy, providing population-level insights into access and patterns of care. Its findings are consistent with international literature describing a “two-tier model” of access, in which diagnosis rather than complex care needs continues to determine eligibility for specialist end-of-life care (Downar et al. 2019). Overcoming this imbalance requires cultural and organizational change through earlier palliative assessment, interdisciplinary coordination, timely referral for non-cancer patients and ensuring hospice policies promote access beyond oncology (Monnery and Droney 2024).

As a descriptive study, this analysis may not capture the full complexity of patient trajectories. Hospice deaths may not represent all hospice patients, as some are discharged to home or hospital, and deaths among those receiving palliative care at home are not included (Cocchi et al. 2024).

## Conclusions

The Italian experience reflects both progress and fragility. The growth of hospice capacity marks an important public health success, but the pandemic setback and the continued predominance of cancer deaths highlight that structural integration is still limited. Continued efforts and research are needed to promote more equitable, diagnosis-independent access to palliative care.

## Data Availability

The data underlying this article were provided by the Italian National Institute of Statistics (ISTAT) under a specific data-use agreement. Access to data is restricted and not publicly available. Derived and aggregated data supporting the findings of this study are available from the corresponding author upon reasonable request and with the permission of ISTAT.
